# Self-directed Learning Theory to Practice: A Footstep towards the Path of being a Life-long Learner

**DOI:** 10.30476/JAMP.2022.94833.1609

**Published:** 2022-07-01

**Authors:** KAILASH CHAROKAR, PUJA DULLOO

**Affiliations:** 1 Department of General Surgery and Medical Education Unit, People's College of Medical Sciences & Research Center, Bhopal, Madhya Pradesh, India; 2 Department of Physiology, Faculty NMC Nodal Center, Pramukhswami Medical College, Bhaikaka University, Karamsad, Gujarat, India

**Keywords:** Self-directed learning, Competency-based education, Curriculum, Assessment, Medical students

## Abstract

The traditional education strategy is insufficient to meet the demands of dynamically changing medical science and the fast-growing medical field. The present Competency-Based Medical Curriculum for medical undergraduates in India emphasizes acquisition of a set of competencies for self-directed learning (SDL) through an explicit approach and dedicated teaching hours in the disciplines which gives the opportunity to develop skills for developing lifelong learners. Self-directed learning cuts across all domains of learning and has a significant potential in shaping transformational learning experiences. The concepts of SDL are based on adult learning principles and experiential learning fostering skills for lifelong learning. In view of the paradigm changes in the new curriculum, it is imperative to understand the basic concepts and the methods for effective practice of SDL in the new curriculum. Faculty development for SDL, ensuring the availability of resources, harnessing the power of information technology, and integrating cognitive and affective assessment strategies enhance the effectiveness of SDL. We revisited the literature, and critically summarize our views on the theory-to-practice concepts of self-directed learning. The article discusses the basic concept of SDL, implementation strategies, and evaluation of self-directed learning.

## Introduction

The medical education system in our country, India, has a new role and a challenge in shaping competent medical professionals to meet the health needs of the rapidly changing society. It is a known fact that the passive unidirectional teaching-learning leads to non-facilitation of meta-cognitive skills, thus causing decreased students’ engagement and motivation towards the course ( 1 ). The traditional teacher-centred curriculum is predominantly focused on knowledge acquisition rather than skills in various learning domains. Therefore, the traditional teaching-focused explicit curriculum limits its scope to transform the learners for the usage of curricular applicability and skill demonstration in a healthcare system. Thus, in an interconnected global community undergraduate medical curriculum should be restructured to contribute to the medical mission with a global outlook and local implementation ( 2 ). There is a need to reorient the medical education curriculum to the prevailing public and societal health needs ( 3 ).

Shifting from a teacher-centered to a student-centric curriculum approach emphasizes deep learning by increasing a sense of learner’s autonomy, reliance on active rather than passive learning, increased responsibility and accountability of the student, and interdependence between teacher and learner, thus developing mutual respect within the learner-teacher relationship, a reflexive approach of part of student and learner ( 4 ). This approach also decreases the learning time duration with few human resources facilities ( 5 ). Therefore, medical education is undergoing a compulsive gradual change to orient and align vibrantly, meaningfully, and purposefully to rise to this occasion. Our medical education is in the process of paradigm change from traditional to outcome-based approach. In the competency-based education, the curriculum is designed on a set of learning outcomes (competency statement) in the beginning, and the teaching-learning-assessment process is aligned to ensure a criterion-based acquisition of these pre-defined competencies. It is not time-bound, and the learner progresses to the next stage in the curriculum only after acquiring the previous one. 

The millennium students equipped with digital technology value planning and shaping their career and making options for their choices for learning more than the earlier generation. Today’s medical students are engaged in learning beyond and outside the classroom, which needs to be welcomed. They navigate a plethora of didactic resources apart from institution-specific materials and select to learn from resources of their choice, so they need to be facilitated rather than taught. In many of the institutes, the medical students no longer attend theory classes in person but rather watch lectures online ( 6 ). According to a survey involving 13,099 US medical students, the percentage of second-year medical students who reported “rarely” attending lectures in person continued to increase, from 26.3% to 37.0% between 2018 and 2020 ( 7 ). This kind of student learning pattern probably implies that they are directing their education by developing and crowd-sourcing didactic resources online and offline beyond the formal curriculum to advance their learning. The medical curriculum needs to align and catch up with the learner-driven evolution in this field of education. Very aptly the Competency-based Vision 2015 document in India has incorporated varied student-centric learning approaches from the first year of medical education which includes Self-directed learning ( 8 , 9 ).

There is an increasingly growing role of medical students in shaping their education and outlining the measures required to ensure that the increase of medical self–directed learning (SDL) by students remains aligned with the educational aims of the medical discipline. SDL fosters and promotes the students’ freedom with autonomy, belongingness with the responsibility of learning, flexibility of learning, and better target-orientedness among the students ( 10 ). “Self-directed learning eventually empowers the medical students to develop the competencies for lifelong learning, which is one of the five roles expected from a Competent Indian medical graduate ( 11 ). The SDL is well supported in the Competency-based curriculum by its student-centrism, foundation course, early clinical exposure, interactive teaching, integrated teaching, strengthening of formative assessments, skills lab teaching, workplace-based clinical teaching and assessment, electives, and academic short-term projects. The SDL approach must be integrated into day-to-day clinical activities nurturing the practice of informal SDL, and in the long run, empowering students in formal SDL.

### Importance of Self-directed learning in the curriculum

“Why” talks about Self-directed learning. Meta-cognition, an unconscious awareness of one’s cognition, is an important requisite of a medical student and includes procedural, declarative, and conditional knowledge ( 12 , 13 ). Self-directed learning necessitates medical students to plan and organize their self-learning and keep task-specific goals in mind ( 14 ). This might facilitate the development of metacognitive skills, transforming the students’ professional identities into effective medical professionals. The proactive learners enter and engage in learning, recall, and apply more purposefully and have greater motivation ( 15 ), so it can be an effective and efficient training approach for medical students. Various studies support the view that SDL is valuable in terms of knowledge acquisition for learning basic medical science content ( 16 , 17 ).

### Definition of Self-directed learning

Malcolm S. Knowles defined SDL as, *“A process in which individuals take the initiative, with or without the help of others, in diagnosing their learning needs, formulating learning goals, identifying human and material resources for learning, choosing and implementing appropriate learning strategies, and evaluating learning outcomes”* ( 18 ). Thus, the students learn on their initiative and have primary responsibility for planning, implementing, and evaluating the effort. The responsibility of learning remains with students ( 19 ). Medical professionals are expected to be self-directed learners for sustaining lifelong learning to keep pace with the dynamically changing science of medicine and technology for their professional self-development. COVID-19 pandemic is a recent example of dynamic changes observed in medical science, digital technology, and educational strategies.

### Theories for SDL

The cognitive learning theory pertains to learning from the processes in the mind (mental and psychological). It involves perceptions, processing of information, and memory in the learning process. It focuses on the point that the students should learn ‘how to learn ( 20 ). Experiential learning occurs when the students reflect on an experience. The student develops a working theory that leads them to an action plan and articulates the plan to variable extents to gain a fresh new experience. The cycle of experiential learning continues. Experiential learning is based on Kolb’s learning cycle ( 21 , 22 ). Humanistic theory is more learner-centered and aims to produce students who have the potential for self-actualization, are self-directed, and are internally motivated ( 23 ). The students plan, conduct, and evaluate their own learning. It has often been described as the goal of adult education emphasizing autonomy and individual freedom in learning ( 24 ).

### Methods of Self-directed learning

The two broad subdivisions of Self-directed learning are facilitated learning and self-paced learning ( 25 ). In the former, the content needs to be delivered through a teacher or a facilitator who communicates with the learner through face-to-face discussion, virtual online, or email posts. On the other hand, self-paced learning needs the learner to be motivated, oriented towards learning, and competent to choose suitable resources for the required content. The curriculum planners recommend that SDL should be utilized frequently to develop lifelong learning skills in medical undergraduates ( 26 ). SDL approach is an option to choose as a teaching method in alignment with some competencies. Therefore, SDL in the curriculum has two components, one as a goal to become a lifelong learner, and the other as a teaching-learning strategy ( 20 ). The prime goal of the SDL is to make students lifelong learners and gain subject topic knowledge as an immediate outcome. 

Some of the approaches to conduct SDL are audio-visual lectures, flipped classrooms, case-based learning, problem-based learning, small group discussion, team-based learning (seminars, journal clubs), open-book examination, and Doughnut Rounds ( 27 ). Doughnut Rounds (DRs) activity enables the students to have a structured conversation with several people in a short space of time ( 28 ). In Doughnut rounds, the students are made to form two concentric circles facing each other. The students interact and communicate on the topic of discussion as were briefed to do so and learn. When the facilitator signals, the inner circle moves clockwise, so the peer partner changes for everyone. Further, the students now exchange views with the new partner. Progressively repeating such steps, each student interacts with all students ( 29 ). One form of SDL exercise practice is to give case-based scenarios and guide the learners with questions, leading them to answers using recommended learning resources ( 30 ). The methods of self-paced learning are online courses, learning management system apps, digital books, assignments, and research projects. 

### Competencies of Self-directed Learners

Knowles (1975) identified the competencies of SDL from the perspective of the individual. The variety of competencies included were understanding the differences between teacher-directed and self-directed learning; determining one’s concept as a self-directed being; relating to peers collaboratively and as resources for learning; diagnosing learning needs and formulating objectives; viewing teachers as facilitators; identifying other resources; and collecting and validating evidence of accomplishments ( 18 ).

For medical students to become self-directed learners, the competencies to be acquired by students, based on our peer group discussion and the articles by Patterson et al. ( 31 ), and Patra, et al. ( 14 ), are: 

identification of the own learning gaps in skills and setting the goal for learning

• self-awareness
• evaluating the human and material resources for learning
• critical thinking
• reflection
• critical appraisal
• information management
• teamwork
• self-evaluation
• peer evaluation

**Figure 1:**
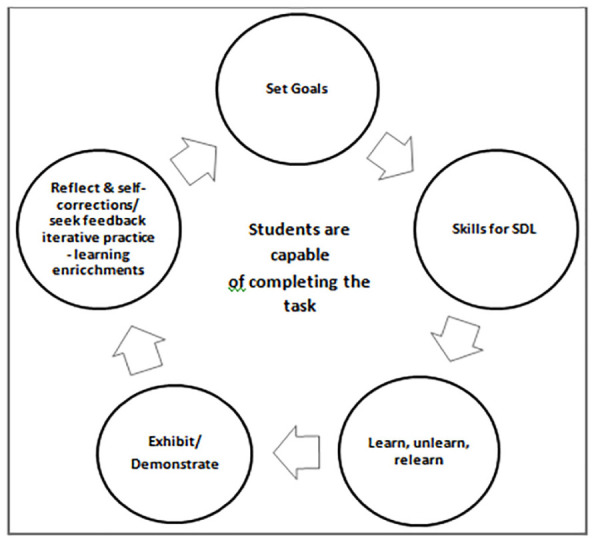
Self Evaluation and Planning of Self Directed Learning

**Figure 2:**
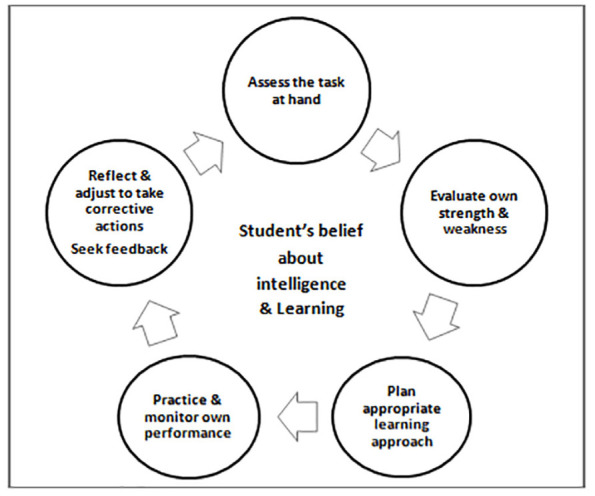
Process of Self Directed Learning

By reflecting on experiences in the curriculum sessions, the students develop competencies of self-directed learning which further leads to acquisition of professionalism. The self-reflection is introspection about how a student navigated and performed in the given tasks, how the experience influenced him, what could have been improved, and how the student would change her/his ways if the similar tasks recur in practice. Each of these competencies are interrelated and influence each other. The students use a set of them and apply for directing and controlling self-learning, self-monitoring, and promoting coperative learning in the group. The students' peer group under the faculty guide is expected to be empathetic to each other, create and sustain friendly learning environments among them, and need to take charge of their learning. The cycle of self-directed learning could be summarized as follows ( 32 ) (Figure 1 and 2).

### The Ascendency of SDL Competencies in the Students

Gerald Grows Staged Self-Directed Learning (SSDL) Model proposes that learners advance through stages of increasing self-direction and that teachers can help or hinder that development. Good teaching matches the learner's stage of self-direction and helps the learner advance toward greater self-direction ( 33 ). The 4 stages of the SSDL Model are: 

**Stage-1:** The student is a ***Dependent learner*** who relies on the teacher, is task-oriented but has no self-direction. The teacher is required to be an authority or a coach. The teacher needs to provide informational lectures, practice drills, coaching with immediate feedback, and overcome deficiencies and resistance. 

**Stage-2:** The student is an ***Interested learner*** who seeks some opportunities, sets some goals, sometimes is directed, and needs confidence to be gained. The teacher needs to be a motivator and guide by giving inspiring lectures, guided discussions, and similar learning strategies with goal setting. 

**Stage-3:** The student is an ***Involved learner*** who identifies learning needs, sets goals for learning, plans actions to achieve goals, and can self-learn. The teacher needs to be a facilitator. The discussions, seminars, and group projects are facilitated by the teacher who participates as an equal. 

**Stage-4:** The students attain the level of ***Self-directed learners***, who are intrinsically motivated, set their own goals, evaluate achievements, and know how to search for valid and reliable resources. Enquiry based learning, Problem based learning, and individual 

assignment/project work, etc are the strategies useful in this stage.

### The Practice of Self-directed Learning

In our view, the stepwise approach for SDL will be a simple guide for facilitating the process of planning, implementing SDL, and assessing the learning outcomes in terms of content and process of the SDL:

1. Training of facilitators for conducting SDL.
2. Assessment of the student’s readiness for introducing the SDL.
3. Selection of the topics from the discipline for conducting SDL.
4. Development of the validated modules for conducting the SDL.
5. Sensitization of the students for SDL which includes group dynamics, search strategies.
6. Provision of the resources needed for implementing SDL.
7. Formation of the students and faculty group.
8. Implementation of the SDL sessions.
9. Assessment of the students for content of topic knowledge, skills, and for the acquisition of SDL competencies.
10. SDL program evaluation: Feedback from the students, teachers, other stakeholders in terms of SDL process evaluation, and SDL outcome evaluation.
11. Performing quality assurance.

### Faculty development for SDL

To introduce new approaches to the students’ teaching-learning-assessments, the capacity building of the faculty is an important positive driver for introducing, sustaining, and consolidating the process of change. Teachers are required to be trained for the preparation of SDL modules, sensitizing students for SDL and learning resource search strategies, and using digital information technology for collaborative group work or independent task completion, assessment strategies for SDL modules, and effective feedback for the same. The preparation of the SDL module is not an individual task of the faculty but of the department as a collaborative output. The Faculty Development Workshops for SDL in the institutes by Medical Education Units/Department, regional, and national CME Workshop needs to conducted at optimum intervals for the capacity building.

### Assessment of student’s readiness for introducing the SDL

Various researchers have developed different self-directed learning readiness scales; Fisher (2009) and Cheng (2010) developed a scale for nursing students, Williamson (2007) for higher education students, and Guglielmino (1977) for medical students. The Guglielmino’s self-directed learning readiness scale (SDLRS) is a self-scoring questionnaire of 58 questions. It has items addressed to personality characteristics, values, attitudes, and skills which shall facilitate SDL ( 34 , 35 ). The instrument measures the factors which include creativity, love for learning, initiative and independence in learning, openness to learning opportunities, informed acceptance of responsibility for one’s learning, self-concept as an effective learner, ability to use basic study, problem-solving skills, and positive orientation to the future. The SDLRS has a test-retest reliability of 0.829 and 0.79, a Pearson split-half reliability estimate of 0.94, and a Cronbach’s alpha reliability coefficient of 0.87 ( 36 ). Another instrument to measure SDL readiness of students is the Self-directed learning instrument (SDLI) scored through a 5-point Liker scale; it consists of 20 items spread over four domains: learning motivation, planning and implementation, self-monitoring, and interpersonal communication. The value of Cronbach's alpha for the total scale reported in the study of Cheng et al. (2010) was 0.916 ( 37 ). The Self-Rating Scale of Self Directed Learning (SRSSDL) was developed by Williamson ( 38 ). It consists of 60 items built on a 5-point Liker scale categorized into five dimensions: awareness (12 items); learning strategies (12 items); learning activities (12 items); evaluation (12 items); and interpersonal skills (12 items). The tool has been subsequently validated in the Italian context by Cadorin and colleagues ( 39 , 40 ). The total score ranges from 40 to 200 and a higher score indicates a higher level of SDL abilities ( 41 ).

One may use any of these scales for assessment of the readiness of the students, or else develop a scale for our Indian students keeping in mind their educational upbringing with learning styles. 

### Selection of the topics from the discipline and development of Module

The topics/competency/learning objectives need to be carefully selected considering that they are relevant, interesting, and motivational for the students. Although it is the learner’s own responsibility to identify the topic, it may not be appropriate for the first year undergraduate students. It may not be possible for a large group of students with a limited faculty number. One must also consider the available time for the topic, so that it is doable. Therefore, the discipline faculty may brainstorm to enlist the topics from the curriculum document. Topics may then be best selected, from the core or non-core topic of the curriculum. It should be clear that those chosen topics should not be retaught. The topic may be introduced with a trigger in the form of a clinical case/audio-video clipping/a topic statement ( 20 ). The task for SDL is usually for a small group ( 8 , 30 ) of students. Having finalized the subject topics, one needs to design a module for the topic contents as per the SDL framework. The milestone of SDL development in the students should be kept in mind according to the stages of Grows model ( 33 ). The session plan needs to be designed, developed, and validated. 

### Students’ sensitization for SDL

The students need to be oriented to basic principles and micromanagement of the SDL program. They need to be sensitized for group dynamics, so that group working leads to team building, interpersonal skill management, conflict management, and the ability to identify their own learning gaps/needs, framing learning objectives for the topic and learning resource search strategies using the MeSH and Boolean operators etc. As they are the main stakeholders and end-users in the education system, they must exhibit belongingness and ownership of their learning which also brings in the responsibility for their learning. 

### Availability of resources needed for implementing SDL

The prime resource for the success of the SDL module is trained facilitators to conduct these sessions. The place of conducting SDL could be classroom/skills lab/department lab/ward side lab or any other suitable venue. Adequately enriched academic and clinical cases as the key for the setting-induction, the digital library, Internet Wi-Fi facilities, audio-visual system recording and playing, Smart Boards, and/or Learning Management system are required. 

### Creating groups for the students and faculty

The student group should be a small group (ideally, 8-10 students per facilitator). The formation of online groups using Google group/ Google classroom, WhatsApp, or any other suitable virtual platform will be required including the faculty guide. An uninterrupted Wi-Fi facilitates the learning as well as the monitoring process during the SDL sessions. One must use computers/Smartphone with the Internet and information technology to continuously improve SDL sessions. During the online engagement, a specific time slot should be assigned to discuss their learning objective parameters. For inculcating the habit of a self-directed learning approach in every student, continuous monitoring of the group dynamics, group discussion and individual participation by the facilitator is required. 

### Implementing the SDL sessions

There are different approaches to implementing the SDL module. Each SDL topic can have two or more contact sessions (online or offline) with an intersession period/gap interval of 7 to 10 days. Each contact session may be of a minimum of 60 minutes and a maximum of 120 minutes ( 20 ). 

*The first contact session* should include a briefing for the session highlighting the topic, the competency to be addressed, and a set induction using a trigger by the facilitator/coordinator. The students be encouraged and given the freedom to express their learning needs, reach a consensus, and formulate the learning objectives within the assigned group. The students take responsibility for their learning. The facilitator can guide the group to refine their learning objectives and identify resources for the topic. The facilitator needs to stimulate the students to think critically by providing a click where and when necessary. 

*The Intersession period:* The duration may be optimum for seven days but should not exceed fourteen days since it will decrease the focus on the SDL topic. The students need to access the resources (textbooks/digital books, e-books, e-journals/recorded audio-visual lectures/Y-tube videos/articles), and self-learn and self-monitor their learning. The faculty guide should be accessible asynchronously online to students in addition to the onsite routine hours. The faculty should also monitor the student’s activities using the online and onsite modes. The facilitator’s role is to monitor the process of group learning, be a guide for resources, and ensure the engagement of every student in the learning process. 

*The second/last contact session* should include an explanation of each learning objective by the participants, which can be through student led-seminars, doughnut rounds, small group discussions, etc., allowing every group member to be an active presenter for his/her learning outcome. 

### Assessment

The assessment strategies for the SDL can be divided into two headings, cognitive gain and the affective attribution skills gain. 

#### Assessing the cognitive academic gain:

classroom/online including MCQs/SAQs test, Quiz, OSCE/OSPE, Structured Oral Viva (SOV), submit assignment/Crosswords, and many more are used.

#### Assessing the SDL competency acquisition:

The Global rating proforma for assessment of competencies of SDL should be used. Self-assessment, the peer assessment, facilitator score sheet on analogue scale or a rubic, the student’s reflection on the SDL experience, log of the visit to the library, log of the visit to e-resources, logbook, portfolio entries for the SDL, all are recommended methods for the assessment of the affective domain of the learner SDL competencies. 

#### Feedback:

One of the major components for improving student learning is based on the immediate feedback given to the student’s group as a whole or individually, depending upon the need of the learner. A summary of the feedback should be given to the students specifying the strength and weaknesses of the group or individual for each session. Peer feedback is an approach for building buddy-community and learning mutually, by taking as well as giving feedback, allowing students to have insight into their way of doing things. It is a learning opportunity for students when they are receptive and will allow them to do better in the future. 

In summary, the ***5 activities*** ( 18 ), during the process of SDL implementation are as follows:

1. Diagnosing the learning gaps - Either the students come up with the learning needs/topics or the facilitator helps to select the topics/competencies.
2. Formulating the learning goals/objectives.
3. Identifying the resource (Human and Material).
4. Choosing and implementing learning strategies.
5. Evaluating the learning outcomes.

SDL is a set of skills that can be taught, learned, and acquired. It is not a teaching strategy, but a philosophy to be imbibed ( 42 ). 

### Program evaluation

This includes the overall SDL program evaluation which may be an objectives-oriented approach (outcomes), and a process-oriented approach. It involves quantitative and qualitative data methods using feedback survey questionnaires from the students, faculty, and other stakeholders. The program evaluation methods may focus on the short-term outcome, mid-term, and long-term longitudinal evaluation of the SDL program. 

### Quality assurance

The report of the program evaluation and the gaps identified in the process of SDL should be analyzed and the recommended corrective measures taken to overcome the issues needed to be addressed. This is further used for future action plans and a continuous improvement in the SDL process in the pursuit of educational excellence and quality assurance. 

### Positive drivers promoting the SDL

In our view, the student-centric curriculum, for the Z/Millennium generation of learners empowered with digital technology, the future relevance of the topics, interesting and thought-provoking set-induction, interactive discussions, student-centric-faculty guide, and effective feedback are some of the strong drivers to promote the SDL. Clinical teaching through case-based learning, problem-based learning, and medical audits based on case records (case-based discussion), clinical reasoning, critical thinking, medical data interpretation, and clinical reasoning models like one minute preceptor, SNAPPS, and Manchester Clinical reasoning tool (MRCT), all lead to the path of SDL ( 43 ). Peine et. al. (2016) concluded in their study that self-directed learning outperformed direct instruction in the environment of a modern, hybrid medical curriculum ( 44 ). Students with characteristics of good engagement in class activities, self-disciplined, organized, and effective communication skills could be academic leaders in promoting the SDL (Table 1). 

**Table 1:** Advantages of Self-directed learning vs. Traditional medical teaching

Self-directed learning	Traditional teaching
**Advantages:**	**Advantages:**
• Learner has the freedom to choose topics, learning resources, time, space, and time management ( 45 ).	• It is teacher controlled.
• It is student-centric.	• It inculcates discipline.
• Learners have freedom to practice their preferred style of learning.	• It has more opportunities for face-to-face interactions with teachers.
• It promotes eLearning.	• It provides more information in a short time frame.
• Deep Learning occurs.	• Influence of teachers on students is higher.
• Team working and Collaborative Learning occur.
• It focus on all the learning domains to be acquired in parallel.
• Information gatherer, interpreter, analyzer, and user thus have a Transformational Learning.
• The target is to be a professional with lifelong learning.
**Disadvantages ( 45 ):**	**Disadvantages:**
• Difficulty in accessing learning resources.	• Predominately unidirectional top-down teaching.
• Difficulty in selecting teaching source.	• Often monotonous.
• Difficulty in accessing tutor/guide.	• Superficial learning.
• Language barrier.	• Focuses predominately on the knowledge domain.
• Time wastage.	• Learning takes place in layers in the stages of the curriculum.
• Difficult to assess the process outcome.	• Lacks transformational power.

### Challenges of SDL

The challenges identified during SDL sessions will be categorized as per student, facilitator, and administrative. The probable challenges which students face are having behavioural barriers (students do not provide appropriate feedback for peers, readiness for SDL), communication barriers (role ambiguity), cognitive and mental barriers due to information overload, lack of focus on learning (mind wandering), last but not the least, environmental barriers in terms of having a heavy workload and inadequate coping skills strategies, and time management techniques.

The Asian countries where traditional teaching has been used for a long time need more support and guidance in the initial years of SDL ( 46 , 47 ). Thus, there is a need for motivating the student by having interactive teaching strategies, effective feedback for their assessment score, and focused mentoring.

To be self-directed, the students essentially need self-belief for SDL, motivation to undertake SDL, and guidance for appropriate soft skills required for SDL ( 48 ). 

The probable challenges from the facilitator’s point of view are limited human resources, resistance in the mind-set of traditional approach facilitators, lack of facilitator training for student-centred approach for teaching learning and assessment, preparing appropriate SDL module which conveys the concept of course topics and skills for providing effective feedback to students and strength to accept feedback from students for further improvement. 

Lastly, administrative challenges identified for conducting SDL are to allocate specific time duration for SDL in a short, fast-paced course, assigning significant weight/age, formative/summative assessment, to the SDL module, appropriate resources (human, infra-structural, or digital) for conducting SDL activities, inability to maintain consistency among facilitators for conducting the SDL session, and inadequate monitoring approaches/skill during the intersession period. 

Everyone will face a challenge of a different type in one or another way and he/she needs to tackle it as per his/her feasibility and environment.

## Conclusion

COVID-19, the pandemic, has taught each one of us a different aspect of life skills, one of which is self-directed learning. Inculcation of this skill within the medical profession curriculum is important due to the fast-pace entry of digital platforms in every field of medicine, say it education, diagnostic tools, patient care, and many more. The teachers need to ensure provision of ample opportunities in formal curriculum to develop the habit of self-directed learning. Literature has shown that the SDL has a significant role to play in developing medical students as “Lifelong Learners”. 

The SDL approach will guide the learners to transform themselves, holistically, by gaining skills of self-evaluation via reflective writing, grading, and learning critical thinking strategies. Further, it will help them to have the team-building capacity, communication skills, conflict management approach, leadership skills, and time management, by receiving inputs from peers and facilitators after SDL sessions. Also, it contributes to development of search strategies to learn the updated concepts and presentation skills by presenting their learning objectives to all within the class during the SDL session.

The key to the successful implementation of SDL lies in developing comprehensive understanding and faculty training programs. The factors like readiness of medical students and availability of material resources, including the teaching resources, are important drivers which significantly influence the success of SDL. The pursuit of SDL leads to continuous professional development in one's own career path. 

## Future Perspective

This study is the initial step for motivating the faculty members, globally, to prepare and implement the SDL module and monitor the outcome of the students in terms of cognitive gain as well as affective attributes via different assessment strategies.

The study would motivate the research to plan a proposal related to the development of the readiness scale for the health professional students in their region/country and tract its outcome in terms of students identifying the need of procuring the skill of self-directed learning. 

Moreover, educationists can undertake a longitudinal study that can provide better statistical data to show how students are performing in the health care system by attaining the skill of SDL specifically by being lifelong learners. The reliability and validity of various assessment strategies need to be identified within the national or international context. The basic layout of the present study, for preparing the SDL module, is to motivate health professionals to plan strategies within their institutes so as to encourage the natural development of self-evaluation initiatives within the students to be lifelong learners. 

## Authors' contribution

K.Ch conceptualized the article, drafted the initial manuscript, flow chart figures 1 and 2, the subsequent edits and revision of the manuscript; and the final manuscript. P.D critically reviewed the initial draft, suggested comments, revisions, and added the texts to the manuscript. All Authors contributed in drafting and revising the manuscript critically for important intellectual content. All authors have read and approved the final manuscript and agree to be accountable for all aspects of the work in ensuring that questions related to the accuracy or integrity of any part of the work are appropriately investigated and resolved.

## Conflict of Interest:

None declared.
